# The Subclonal Architecture of Metastatic Breast Cancer: Results from a Prospective Community-Based Rapid Autopsy Program “CASCADE”

**DOI:** 10.1371/journal.pmed.1002204

**Published:** 2016-12-27

**Authors:** Peter Savas, Zhi Ling Teo, Christophe Lefevre, Christoffer Flensburg, Franco Caramia, Kathryn Alsop, Mariam Mansour, Prudence A. Francis, Heather A. Thorne, Maria Joao Silva, Nnennaya Kanu, Michelle Dietzen, Andrew Rowan, Maik Kschischo, Stephen Fox, David D. Bowtell, Sarah-Jane Dawson, Terence P. Speed, Charles Swanton, Sherene Loi

**Affiliations:** 1 Division of Research, Peter MacCallum Cancer Centre, Melbourne, Victoria, Australia; 2 The Walter and Eliza Hall Institute of Medical Research, Parkville, Victoria, Australia; 3 Faculty of Medicine, Dentistry and Health Sciences, The University of Melbourne, Victoria, Australia; 4 Cancer Genomics Program, Division of Research, Peter MacCallum Cancer Centre, Melbourne, Victoria, Australia; 5 Division of Cancer Medicine, Peter MacCallum Cancer Centre, Melbourne, Victoria, Australia; 6 kConFab, Division of Research, Peter MacCallum Cancer Centre, Melbourne, Victoria, Australia; 7 UCL Cancer Institute, CRUK Lung Cancer Centre of Excellence, London, United Kingdom; 8 The Francis Crick Institute, London, United Kingdom; 9 University of Applied Sciences Koblenz, RheinAhrCampus Remagen, Department of Mathematics and Technology, Remagen, Germany; 10 Department of Pathology, Peter MacCallum Cancer Centre, Melbourne, Victoria, Australia; 11 Sir Peter MacCallum Department of Oncology, the University of Melbourne, Victoria, Australia; 12 Bioinformatics Division, Walter & Eliza Hall Institute of Medical Research, Parkville, Victoria, Australia; 13 Department of Mathematics and Statistics, University of Melbourne, Parkville, Victoria, Australia; MSKCC, UNITED STATES

## Abstract

**Background:**

Understanding the cancer genome is seen as a key step in improving outcomes for cancer patients. Genomic assays are emerging as a possible avenue to personalised medicine in breast cancer. However, evolution of the cancer genome during the natural history of breast cancer is largely unknown, as is the profile of disease at death. We sought to study in detail these aspects of advanced breast cancers that have resulted in lethal disease.

**Methods and Findings:**

Three patients with oestrogen-receptor (ER)-positive, human epidermal growth factor receptor 2 (HER2)-negative breast cancer and one patient with triple negative breast cancer underwent rapid autopsy as part of an institutional prospective community-based rapid autopsy program (CASCADE). Cases represented a range of management problems in breast cancer, including late relapse after early stage disease, de novo metastatic disease, discordant disease response, and disease refractory to treatment. Between 5 and 12 metastatic sites were collected at autopsy together with available primary tumours and longitudinal metastatic biopsies taken during life. Samples underwent paired tumour-normal whole exome sequencing and single nucleotide polymorphism (SNP) arrays. Subclonal architectures were inferred by jointly analysing all samples from each patient. Mutations were validated using high depth amplicon sequencing.

Between cases, there were significant differences in mutational burden, driver mutations, mutational processes, and copy number variation. Within each case, we found dramatic heterogeneity in subclonal structure from primary to metastatic disease and between metastatic sites, such that no single lesion captured the breadth of disease. Metastatic cross-seeding was found in each case, and treatment drove subclonal diversification. Subclones displayed parallel evolution of treatment resistance in some cases and apparent augmentation of key oncogenic drivers as an alternative resistance mechanism. We also observed the role of mutational processes in subclonal evolution.

Limitations of this study include the potential for bias introduced by joint analysis of formalin-fixed archival specimens with fresh specimens and the difficulties in resolving subclones with whole exome sequencing. Other alterations that could define subclones such as structural variants or epigenetic modifications were not assessed.

**Conclusions:**

This study highlights various mechanisms that shape the genome of metastatic breast cancer and the value of studying advanced disease in detail. Treatment drives significant genomic heterogeneity in breast cancers which has implications for disease monitoring and treatment selection in the personalised medicine paradigm.

## Abstract 2

Sherene Loi and colleagues describe the mutational landscape of breast cancer metastases at death, providing key insights into this disease at its lethal phase.

## Author Summary

**Why Was This Study Done?:**

- We understand very little about the genomic changes that take place in advanced breast cancers, particularly in the most advanced disease that results in death.
- Seeking this information is expected to provide important insights into cancer biology and help us understand how advanced breast cancer evolves over time and the impact this has on the natural history of the disease.

**What Did the Researchers Do and Find?:**

- We conducted rapid autopsies on four patients that died of advanced breast cancer and extensively profiled multiple lesions, which were compared to biopsies taken whilst patients were alive, where possible.
- We aimed to reconstruct the relationship between different metastatic lesions and understand the tumour cell subpopulations that make up metastatic lesions.
- Our findings reveal significant heterogeneity and evolution over time, which was shaped by treatment, mutational processes, and the interaction of these factors with alterations in cancer-promoting driver genes.

**What Do These Findings Mean?:**

- Our findings reveal the complexity of the cancer genome in advanced disease and highlight the importance of ongoing monitoring and reassessment of the genomic profile if this is to be used to guide therapy.
- Detailed autopsy studies in small numbers of patients can yield valuable insights into cancer biology.
- Detecting small populations of cancer cells from actual patient specimens is difficult, and the findings in this study could be limited by poor sensitivity to detect rare populations.

## Introduction

Heterogeneity in the natural history of advanced cancers has long been noted. The advent of cancer genomics has revealed that significant heterogeneity may exist both between and within lesions in the same patient. Since that time, autopsy studies have found a great variety of genomic heterogeneity in multiple cancer types. Evolution over time has also been documented, with varying influences of therapy in shaping the subclonal architecture of advanced disease. At the same time, the clinical significance of heterogeneity is yet to be established.

Patients die of metastatic disease, but little is known about the biology of this late-stage, lethal process. Genomics is a mature and robust platform for querying this biology. In breast cancer, although many thousands of primary tumours have been characterised in detail, such comprehensive data do not exist for metastatic disease, particularly in the most advanced and lethal disease. Shah et al. studied genomic heterogeneity in a case of lobular breast cancer that recurred 9 years after initial diagnosis. This single case showed genomic evolution over time from primary to metastatic disease [1]. Ding et al. analysed a chemoresistant metastatic basal-like breast cancer and showed low divergence between primary and metastatic lesions, with 48 of 50 mutations in common between sites [2].

More recently, the focus has shifted to understanding heterogeneity at the subclonal level. Complex but reproducible subclonal dynamics were revealed by Eirew et al. in a large study of treatment-naïve breast cancer xenografts analysed at the single-cell level [3]. Murtaza et al. combined multi-region sequencing at autopsy with circulating DNA (ctDNA) measurements, finding that ctDNA captured some of the underlying subclonal dynamics [4]. Juric et al. used sequential biopsies and samples from autopsy to study the evolution of resistance to a PI3K inhibitor in a case of hormone-positive breast cancer, finding evidence for multiple resistance mechanisms evolving simultaneously in spatially distinct sites (termed convergent evolution) [5]. Yates et al. performed a combination of whole genome and targeted sequencing on 50 breast cancer cases and inferred subclonal populations, finding variable intra-lesional heterogeneity [6]. Low prevalence or “minor” subclones in primary tumours could be seen giving rise to recurrent disease and convergent evolution occurred both late and early in the disease course. The profound influence of treatment on the subclonal structure of breast cancer has also been delineated in human epidermal growth factor receptor 2 (HER2)-positive tumours using a method that visualises driver mutation heterogeneity in different tumour subpopulations [7]. This study also found an association between heterogeneity and poor clinical outcome. Determining the genomic changes that define subclonal populations allows the evolutionary history of a cancer to be inferred, as was performed by Gao et al. in a single-cell whole genome sequencing study of triple negative breast cancers [8]. Well-demarcated subclonal populations could be defined by copy number alterations (CNA) without the presence of populations showing intermediate copy number states. These findings are most consistent with punctuated evolution of neoplastic phenotypes early in the natural history of a tumour rather than gradual accumulation of genomic alterations. These studies paint an expanding picture of the challenges and opportunities in understanding the complexities of advanced disease. Many questions remain about the relationship between breast cancer’s capacity to evolve towards heterogeneous states and clinical outcome.

To understand the biology of lethal metastatic disease, our institution has implemented a prospective community-based rapid autopsy program (CASCADE) in which patients consent whilst alive to allow tissue donation after death [9]. In this study, we attempted to understand the evolutionary history of four patients with lethal breast cancer who participated in the CASCADE program: an aggressive and treatment-resistant triple negative breast cancer where the patient died 12 months from diagnosis (TN1); an oestrogen-receptor (ER)-positive HER2-negative breast cancer with late relapse 7 years from diagnosis of early-stage disease (ER1); a de novo metastatic ER-positive, HER2-negative breast cancer (ER2); and an ER-positive, HER2-negative breast cancer in a young patient with multiple instances of discordant responses to treatment between metastatic lesions (ER3). By interrogating the cancer genome at multiple metastatic sites, including metastatic biopsies taken during life, we aimed to infer the subclonal structure and evolutionary history of the disease for each case as it progressed from the primary tumour to lethal metastatic dissemination.

## Methods

As breast cancer is relatively treatment responsive compared to other tumour types, we focussed our analysis on understanding subclonal composition and how this evolves over time under the influence of therapy. The first case was recruited in 2013, at which time methods for performing subclonal inference on multiple samples were not mature. By the time the final case was recruited in 2015, the field had advanced considerably, which made this approach feasible, as will be detailed below. All four cases were analysed concurrently.

Patients were recruited by their treating clinicians to CASCADE, a prospective community-based rapid autopsy program [9] conducted in association with the Kathleen Cuningham Foundation Consortium for Research into Familial Breast Cancer (kConFab) and approved by the Human Research Ethics Committee of the Peter MacCallum Cancer Centre, Melbourne (HREC approval numbers: CASCADE 13/122, kConFab 92/97 and 11/102). Any patient with advanced breast cancer was eligible. All patients provided written informed consent.

### Study Procedures

**Fig 1:**
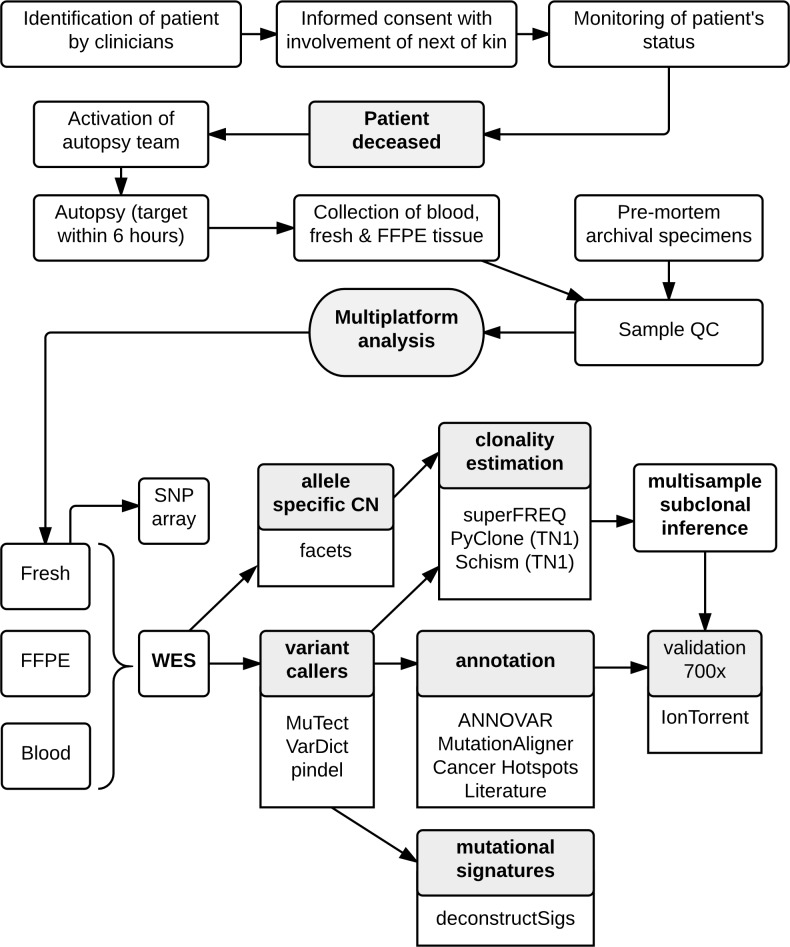
Workflow diagram giving overview of methodology.

The workflow for the CASCADE program is shown in Fig 1. Once recruited, the CASCADE coordinator communicates with the patient, their family, and the associated health care providers regarding the patient’s status and ongoing willingness to participate. Following the patient’s death, the next of kin specifies when the body can be transferred for autopsy. Autopsies were conducted by a specialist forensic pathologist assisted by the research team. Tumour tissue was harvested from multiple representative metastatic sites. Each lesion was divided into portions that were immediately snap frozen in liquid nitrogen and also fixed in formalin for subsequent paraffin embedding. Frozen samples were used for sequencing where possible. Prospectively collected fresh frozen samples were available for some cases. For all fresh tissues, frozen sections were reviewed by a pathologist to confirm the presence of tumour, quantify necrosis, and estimate tumour cellularity. Premorbid formalin-fixed, paraffin-embedded (FFPE) archival samples from primary tumours and metastatic lesions were also obtained.

### Whole Exome Sequencing

DNA was extracted from sections cut from frozen tissues or FFPE blocks. For three of the cases, ER1–ER3, whole exome libraries were prepared using the Roche-NimbleGen SeqCap EZ exome version 3. For case TN1, libraries were prepared using the Illumina Nextera Rapid Exome. All libraries were sequenced on the Illumina Hiseq platform. TN1 had DNA submitted for single nucleotide polymorphism (SNP) array analysis with the Illumina Human Omni 2.5S beadchip, and other cases had DNA submitted for SNP array analysis on the Affymetrix Genome Wide SNP 6.0 array. Blood was available for all cases for reference germline DNA.

After adapter trimming with cutadapt [10], raw sequence data were aligned to the human reference genome GRCh37 with BWA [11]. Two samples for TN1 exhibited low sequencing yield and a large number of likely artefactual mutations due to FFPE processing, and were excluded from further analysis. Fresh frozen samples were sequenced to a mean depth of 105, with FFPE samples sequenced to a mean depth of 75. Following alignment, BAM files were processed according to GATK best practices [12]. Variants were called using MuTect [13] with default parameters. Indels were called with VarDict [14] and pindel [15].

Called variants were annotated with ANNOVAR [16]. Variants were filtered as follows: exclusion of variants in 1000 Genomes and the Exome Sequencing Project [17,18]; variants with an allele frequency ≥2% or with 2 or more supporting reads in the normal; and allele frequency <5% in fresh tissues or <10% in FFPE tissues. Indels were manually reviewed and excluded if adjoining >4 homopolymer runs or repetitive regions.

Copy number was called from whole exome sequencing (WES) data using the facets package, which also provided purity and ploidy estimates [19]. For the Illumina bead chips, raw data were tQN normalised [20], and segmented allele-specific copy number was called using OncoSNP [21]. For the Affymetrix SNP chips, samples were processed using the Aroma package, applying TumorBoost and PSBCS [22,23]. Output of facets was compared to the SNP chip data, and, in some cases, the parameters were adjusted to exclude less likely combinations of copy number and purity. As SNP arrays were not available for the FFPE samples, whole exome copy number was used for all analyses. Copy number segmentation determined from SNP arrays was used as orthogonal validation of WES copy number calls from facets.

To determine the functional significance of novel nonsynonymous variants, the following steps were taken: search COSMIC [24] and TCGA [25] for previous reports (across all tumour types); prioritise variants with a high CADD score [26]; map variants to functional domains of the gene, if possible; check if variant falls in a mutational hotspot using MutationAligner [27] and Cancer HotSpots [28]; and review literature (using GeneRIF queried via MyGene.info [29]). Annotation of CNA was limited to genes found to be recurrently altered in the literature [30,31].

### Subclonal Inference

superFREQ is a cancer exome clonality inference tool that takes advantage of multiple samples from the same individual by tracking both single nucleotide variants (SNV) and CNA across samples [32]. This allows the detection of highly subclonal somatic mutations that are present at higher cell fraction in other samples. An advantage of superFREQ is that it estimates and propagates statistical and systematic error sources throughout the analysis, thus decreasing the number of false positives and allowing downstream analysis of uncertainties. It also accepts aligned sequence data as input directly. Briefly, the pipeline performs the following steps: (1) GC bias correction, (2) differential coverage analysis with limma-voom [33], (3) examines variant positions shared between individual samples, flagging variants for base quality, mapping quality, strand bias, stuttering, or other artefacts detected in the pool of normal control samples, (4) summarises differential coverage and SNPs for each gene, (5) recursively clusters neighbouring genes with sufficiently similar differential coverage or SNP frequencies until a segmentation of the genome is achieved for each sample, (6) summarises consensus coverage and SNP frequencies for each segment and renormalizes, taking accuracy of coverage and SNP frequency into account, (7) calls CNAs and clonality in each segment based on coverage and SNP frequency, (8) calculates clonalities of somatic SNVs using local CNA, (9) tracks clonalities of SNVs and CNAs over multiple samples to determine if the same or different alleles are gained/lost between samples, (10) clusters mutations (SNVs or CNAs) with similar clonalities in all samples into clones, (11) sorts clones into a tree structure, with smaller clones being assigned as subclones when the sum of the clonalities of disjoint subclones cannot be larger than the clonality of the containing clone. Not all mutations are allocated to subclones by superFREQ, usually due to highly deranged copy number, which does not permit the cancer cell fraction to be reliably determined. Others have noted this problem with subclonal reconstruction [6].

Case TN1 displayed a high degree of copy number variation, including large swathes of loss of heterozygosity (LOH) affecting over 50% of the genome. Accurate clonal inference with superFREQ was not successful. As an alternative, mutation clonality was determined using PyClone [34], with allele-specific copy number provided by the R package facets [19]. Mutation genotypes were built using “parental_copy_number” mode. For mutations in regions of LOH, genotypes consistent with LOH were assigned a prior weight of 10, and other genotypes down weighted to 0.1. All other genotype prior weights were left as 1. From the PyClone output, the median clonality for each mutation (or cancer cell fraction) was clustered via affinity propagation using the Schism package [35]. Mutation clusters were reviewed to separate agglomerated private subclones if needed, and then the reviewed clusters were supplied to Schism’s genetic algorithm to construct a multi-sample phylogeny. To validate this approach, high depth validation sequencing of mutations found in more than one sample was subjected to the same process to check the consistency of the tree with the WES data.

### Validation

From each case, mutations and indels were selected for high depth validation based on their contribution to the subclonal phylogeny and biological interest. An Ion AmpliSeq custom panel was designed for the variants of interest in each case, and multiplex PCR was performed as per the manufacturer’s protocol. Libraries were sequenced on the Ion Torrent PGM sequencer (Life Technologies) to a median depth of 770. Wild-type and mutant reads from validation loci were extracted from BAM files using the GenomicAligments R package [36]. Taking loci where there were at least 20 reads, a mutation was considered validated if there was a significant deviation from an expected error rate of 1/200 using the p-binomial test [1].

### Mutational Signatures

Mutational signatures were ascertained in a two-step process. Somatic mutations were filtered to have allele frequency >10% in FFPE samples to avoid detecting formalin fixation artefact and >5% in fresh samples. To increase detection power and avoid spurious signature detection, all unique mutations from each patient were pooled together and used as input to deconstructSigs [37]. “default” normalisation was used as per the authors’ instructions, with a minimum signature contribution of 0.06. The 30 signatures found in the latest COSMIC classification were used [38,39]. The contribution of the restricted set of signatures found in the pooled mutation set was then examined in each sample. To test the robustness of signature detection, pooled mutations were randomly downsampled to between 10% and 90% of the original mutations, and deconstructSigs was re-run on 1,000 such downsampled sets.

### ctDNA and Immunohistochemistry

ctDNA was assayed as previously described [40]. For immunohistochemistry, heat-induced antigen retrieval was performed in 1× citrate buffer (Thermo Scientific). Samples were blocked in 2% bovine serum albumin in tris-buffered saline/Polysorbate 20 solution and endogenous peroxidase inactivated in 1.5% H2O2. Samples were incubated with primary antibodies, including AGF2 (Abcam), CHD4 (Abcam), and Cullin1 (Cell Signalling Technology). Biotinylated species-specific secondary antibodies were used at 1:300 (Dako) followed by the avidin-biotin-complex (ABC) method prior to visualisation with 3,3′-Diaminobenzidine (DAB) chromogen (Dako). Bright-field microscopy was performed on an Olympus BX-51 microscope. Murine inguinal mammary fat pads were used as normal control tissue.

## Results

Three ER-positive cases (denoted ER1, ER2, and ER3) and one triple negative case (denoted TN1) were analysed. Cases were recruited sequentially, and no cases were excluded from the analysis. In all cases, primary tumours were available as FFPE samples. Original pathology reports describing the macro-dissection of the primary tumours were used to select FFPE blocks in spatially distinct regions for each patient. In ER2, ER3, and TN1, metastatic biopsies taken during life were also available for analysis, as well as ctDNA for ER2. To study heterogeneity and evolution in detail, 8 (ER1), 13 (ER2), 16 (ER3), and 15 (TN1) samples were sequenced from each patient. Samples are summarised in S1 Table.

Patient TN1 was a woman diagnosed with a locally advanced triple negative breast cancer not associated with a *BRCA1* or *BRCA2* germline mutation, at age 39. She had a clinical response to a third-generation standard adjuvant chemotherapy regimen and underwent mastectomy, which showed significant residual disease. Shortly thereafter, she developed a solitary bony metastasis, followed by a liver metastasis and then multiple additional sites of metastatic disease across lung, liver, and brain before dying of liver failure less than 12 months from first diagnosis. She was treated with trastuzumab briefly when the breast primary was found to have focal HER2 positivity, which was not seen again in other lesions. In contrast, patient ER1 relapsed with metastatic disease 7 years after her primary diagnosis at age 42 and underwent a series of endocrine therapies before developing liver metastases and dying of liver failure after surviving 8 years with metastatic disease. Patient ER2 had de novo metastatic disease diagnosed at age 35 and survived 3 years, receiving multiple lines of chemotherapy and endocrine therapy. Patient ER3 did not receive surgical or medical treatment after initial diagnosis of early stage disease at age 36 due to personal circumstances. She presented 18 months later with metastatic disease and displayed discordant responses to therapy during her disease course, which ran 4 years in duration before death.

Of 52 samples, three samples from TN1 were sequenced but not used in further analysis, as they failed quality control (a breast core biopsy and biopsy of a femoral lesion taken premortem, and a subcarinal lymph node from autopsy). One additional sample, a brain metastasis from TN1, failed to sequence. Quality control metrics along with per sample validation rates are shown in S2 and S3 Tables. For mutations detected with an allele frequency ≥10% from WES, high depth validation rates for FFPE samples ranged from 95%–100% and from 97%–100% for fresh samples. When mutations with an allele frequency ≥5% were used, 44/45 samples had validation rates of 95% or greater, with one sample from TN1 having a rate of 94%. Importantly, high depth orthogonal validation did not affect the structure of the subclonal tree. Three samples could not undergo validation due to insufficient DNA (1 pre-chemotherapy breast biopsy for ER2 and the 2 primary samples for ER3).

### Subclonal Architecture Defines Metastatic Spread

**Fig 2:**
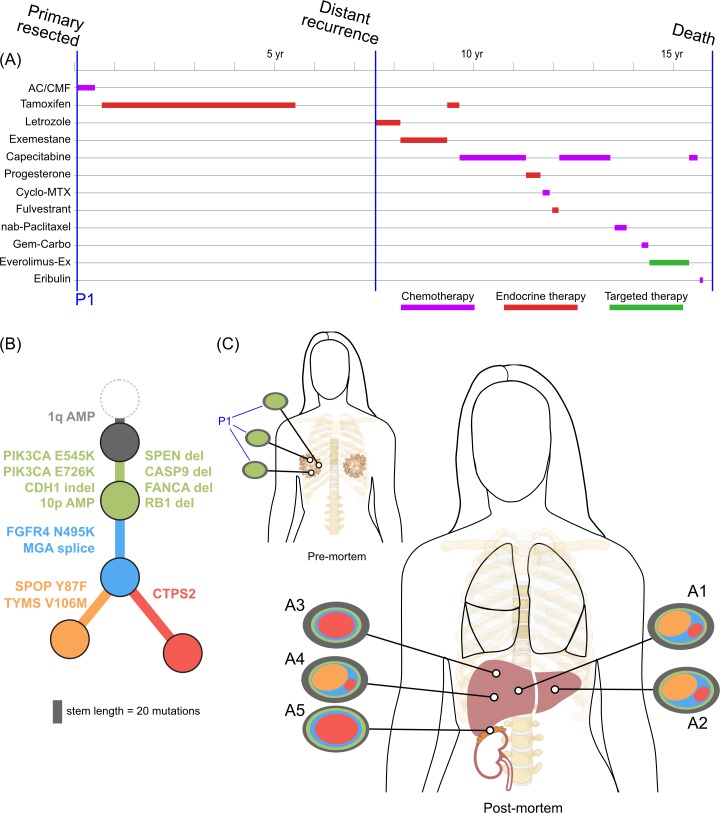
**Treatment history (A) and subclonal structure (B and C) for ER1, inferred by superFREQ.** Pre- and postmortem samples are shown. AC: doxorubicin, cyclophosphamide; CMF: cyclophosphamide, methotrexate, 5-fluorouracil; Cyclo-MTX: cyclophosphamide, methotrexate; Gem-Carbo: gemcitabine, carboplatin; Everolimus-Ex: everolimus, exemestane. P1: multiple samples from archival breast primary; A1, A3, A4: right liver lobe metastases; A2: left liver lobe metastasis; A5: right adrenal lesion.

**Fig 3:**
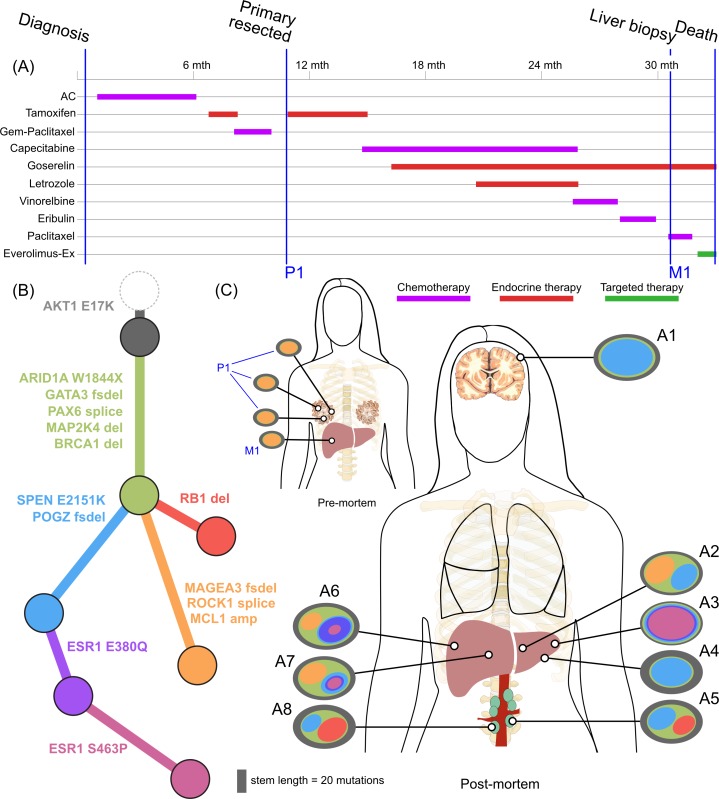
**Treatment history (A) and subclonal structure (B and C) for ER2, inferred by superFREQ.** Pre- and postmortem samples are shown. AC: doxorubicin, cyclophosphamide; Gem-Paclitaxel: gemcitabine, paclitaxel; Everolimus-Ex: everolimus, exemestane. P1: multiple samples from archival breast primary, including pretreatment breast core biopsy; M1: liver core biopsy; A1: dural metastasis; A2, A3, A4: left liver lobe metastases; A5, A8: para-aortic nodal metastases; A6, A7: right liver lobe metastases.

**Fig 4:**
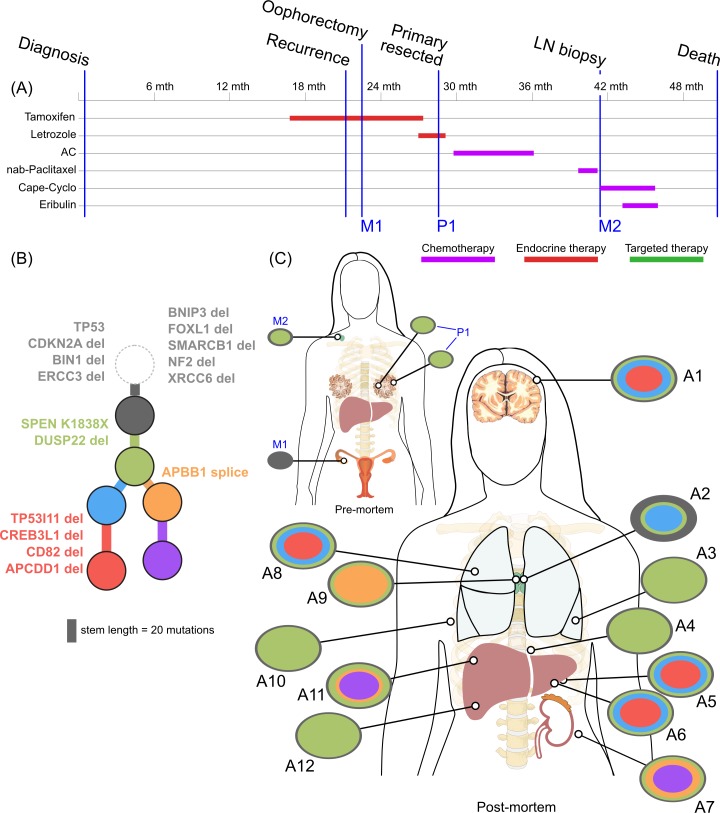
**Treatment history (A) and subclonal structure (B and C) for ER3, inferred by superFREQ.** Pre- and postmortem samples are shown. Cape-Cyclo: capecitabine, cyclophosphamide; P1: multiple samples from archival breast primary; M1: ovarian metastasis; M2: supraclavicular lymph node biopsy; A1: brain metastasis; A2, A9: left and right hilar lymph nodes; A3: left lung lower lobe metastasis; A4: left paravertebral soft-tissue metastasis; A5, A6: anterior and posterior left liver lobe lesions; A7: perinephric metastasis; A8: right upper lobe lesion; A10: rib metastasis; A11, A12: right liver lobe metastases.

**Fig 5:**
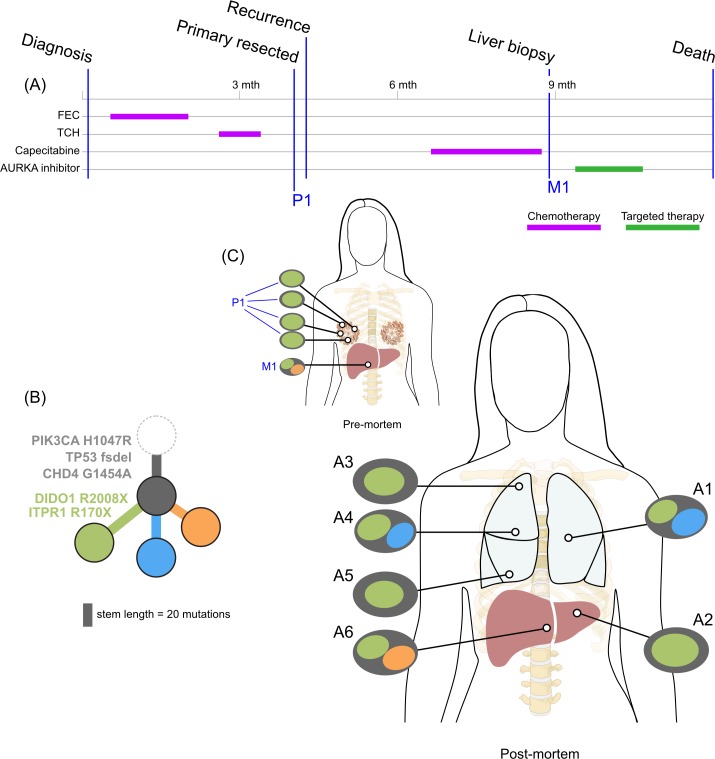
**Treatment history (A) and subclonal structure (B and C) for TN1, inferred using PyClone and Schism.** Pre- and postmortem samples are shown. FEC: 5-fluorouracil, epirubicin, cyclophosphamide; TCH: docetaxel, carboplatin, trastuzumab; AURKA: aurora kinase; P1: multiple samples from archival breast primary (post-neoadjuvant chemotherapy); M1: liver core biopsy; A1: left upper lobe metastasis; A2: left liver lobe metastasis; A3, A4, A5: left lung metastases; A6: right liver lobe metastasis.

Inferring subclones and constructing subclonal phylogenies is a complex problem, requiring high sequencing depth and/or multiple samples to increase sensitivity and reduce the space of possible tree reconstructions [41–43]. By utilising multiple samples from each patient, we were able to construct consistent subclonal phylogenies using mutation and copy number information (Figs 2–5). Private subclones (that is, subclones that are found only in one lesion) are not displayed in these figures for clarity, as they do not contribute to the core tree structure; therefore, all clones displayed were found in at least two spatially distinct sites.

All ER-positive cases had founding clones containing known driver alterations. These founding clones developed secondary alterations before giving rise to diverse subclones across multiple metastatic sites. By way of example, in ER1 shown in Fig 2B, we inferred the presence of an intermediate blue clone not found in isolation. This follows from (1) the absence of this clone in the primary tumours, indicating the blue clone is a true subclone; (2) the absence of the orange subclone in two samples where the red subclone has a high clonality, indicating the red subclone did not arise in the orange subclone; and (3) two different subclones (red and orange) with the blue clone as an ancestor. This intermediate blue clone was marked by an *FGFR4* tyrosine kinase domain mutation and a splice site mutation in *MGA*, which counter-regulates *MYC* activity. The blue clone also shows deletions in genomic regions containing known tumour suppressor genes *RB1*, *SPEN*, *CASP9*, and *FANCA*. These mutations and CNAs likely conferred metastatic potential, as all subsequent metastatic subclones arose from this intermediate clone, as described above. The *FGFR4* mutation could not be detected in three spatially separated samples of the primary tumour, even with high depth validation sequencing, although this does not rule out very low prevalence subclones at the time of diagnosis. Emergence of an unheralded “lethal subclone” following treatment has been documented in many tumour types, including breast cancer, but here it associated with delayed disease relapse.

**Fig 6:**
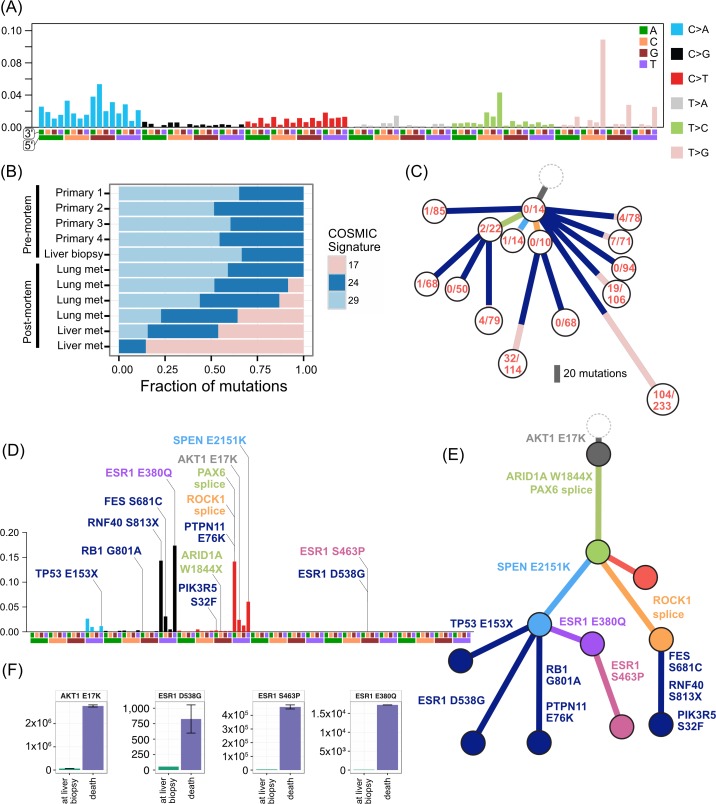
Mutational signatures in TN1 and ER2, and ctDNA assays in ER2. (A) Reconstruction of mutational context plot with deconstructSigs for TN1 using COSMIC signatures 17, 24, and 29. 5′ and 3′ nucleotides are indicated by colour code on *x*-axis. (B) Contribution of each signature per sample. (C) Subclonal phylogeny for TN1 showing private and public subclones. Private subclones in dark blue. Mutations arising from signature 17 represented in pink along branches. (D) Reconstruction of mutational context for ER2 using APOBEC signatures 2 and 13 detected with deconstructSigs, with origin of key mutations overlaid. Colour codings as for (A). (E) Subclonal phylogeny for ER2, with private subclones in dark blue. (F) Bar charts show ctDNA results at time of liver biopsy and later at death. *y*-axis unit is copies per millilitre.

ER2 and ER3 display a different relationship between subclonal structure and metastatic disease. For ER2, a biopsy of the breast mass was the index lesion, and the breast primary was removed after chemotherapy, followed by a liver biopsy after several lines of therapy. All premortem samples displayed a similar subclonal structure, with a subclone (orange) marked by a frameshift deletion in the cancer testis antigen *MAGEA3* and a splice site mutation in *ROCK1* that was subsequently detected at multiple sites in the liver at autopsy. The absence of this subclone at other metastatic sites, however, shows there was early divergence and parallel evolution of at least two other subclones (blue and red). ctDNA for the *AKT1* (E17K) mutation and three *ESR1* mutations (D538G, S463P, E380Q) were assayed in plasma taken at the time of liver biopsy and subsequently after death. Fig 6F shows all these mutations were detectable at both time points (albeit at very low levels for the first time point), even though the liver biopsy WES data did not reveal any *ESR1* mutations.

ER3 was a case of delayed presentation, with 2 years elapsing between initial detection of early stage disease and subsequent metastatic disease, without intervening treatment. No samples could be obtained from the original diagnosis. In a similar fashion to ER1, the primary subclones have a linear monophyletic relationship with the metastatic subclones. This case is distinguished, however, by an ovarian lesion that contains the founding clone only. This ovarian lesion was discovered incidentally during a therapeutic oophorectomy prior to any anticancer therapy. Hence, despite lacking the more complex subclonal structure seen in other lesions, this disease possessed the ability to metastasise very early. Whether this ovarian metastasis would have resulted in clinically significant disease is unknown. The rest of the lesions must have arisen from dissemination of a different subclone, which had acquired the *SPEN* K1838X nonsense mutation and deletion of the negative modulator of oestrogen signalling *DUSP22*. These alterations occurred while the patient was receiving endocrine therapy.

TN1 displayed both short linear evolution and early divergence. Fig 5 shows the subclonal structure of the primary is found in all metastatic lesions via the green clone, but the blue and orange clone diverged from a common ancestor derived from an earlier progenitor of the green clone. Fig 5C shows the private subclones, some of which also emerged from the common ancestor. It is noteworthy that this case, with the most aggressive disease history with no response to three different standard chemotherapy regimens, had the fewest common subclones. S1 Fig demonstrates that CDH4, which contains a truncal mutation, is expressed at the protein level.

A key question is whether metastatic disease from one lesion can cross-seed anatomically distant sites. This is difficult to distinguish from a subclinical low prevalence clone seeding two different metastatic sites, a disseminative pattern rather than cross-seeding. All cases showed spatially distinct lesions with very similar subclonal structures (e.g., liver lesions in ER1; para-aortic nodal metastases in ER2; brain, lung, and liver metastases in ER3; lung metastases in TN1). This is suggestive of metastatic cross-seeding. In addition, ER1, ER2, and TN1 showed recurrent subclonal mixtures, raising the possibility of seeding by polyclonal clusters sustained by clonal cooperation, in which two subclones help maintain each other’s survival and confer novel phenotypic traits [3,44]. For such patterns to arise from dissemination alone rather than cross-seeding, polyclonal seeding, or cooperation, multiple waves of dissemination by different subclones from a primary tumour to the same metastatic site would be required.

### Copy Number and Subclonal Evolution

**Fig 7:**
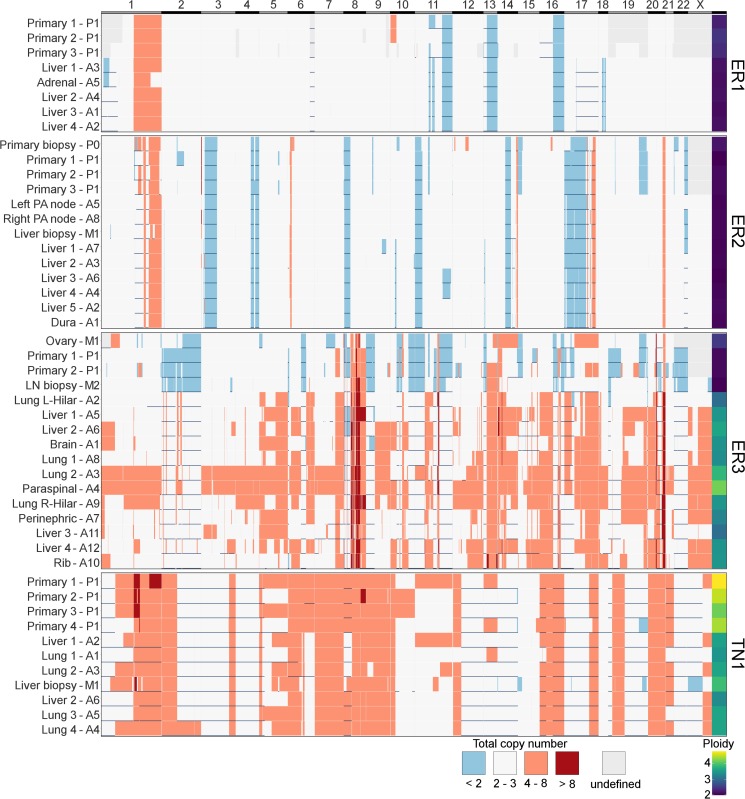
Heatmap of copy number across all samples. *x*-axis shows all coding genes in order of chromosomal coordinate. Grey areas on map represent difficulty in calling allele-specific copy number in FFPE samples. Horizontal blue lines indicate areas of LOH. Deep red is high level amplification, pale red is amplified with four or more copies, and blue is deleted with less than two copies. White is two to three copies. Ploidy is displayed in last column on right. *y*-axis terms correspond to Figs 2–5.

In contrast to subclonal mutations, copy number changes between samples were relatively stable (Fig 7) for ER1, ER2, and TN1. Areas of LOH were particularly consistent across samples. ER1 and ER2 displayed relatively few CNA, with overall 80%–85% of coding genes unaffected. ER3 showed widespread copy number derangement affecting 40%–60% of coding genes, particularly on chromosomes 8 and 20. The ploidy of ER3 increased from early to late stage disease; the primary, ovarian metastasis, and lymph node biopsies all displayed diploid genomes, with autopsy samples showing triploidy and tetraploidy in some cases (mean ploidy across samples 3.2). There was inter-lesion copy number heterogeneity for ER3. This is reflected in the relative abundance of subclonal copy number events in ER3 called by superFREQ, with a mean of 22 copy number events per subclone for ER3, compared to 7 and 11 for ER1 and ER2, respectively. Chromosomal instability (CIN) was therefore an ongoing process contributing to subclonal evolution in ER3. TN1 showed the most deranged genome, which also had a mean ploidy of 3.9, consistent with genome doubling, with 40%–50% of genes subject to LOH and some form of copy number derangement affecting 70%–80% of genes. Subclonal copy number could not be called satisfactorily for TN1. Although less deranged, in ER1, there was evidence that the primary event was amplification of 1p, prior to driver mutations in *PIK3CA* (Fig 2B).

These findings are consistent with the growing body of work that extensive CIN takes place early in the evolution of a tumour. In particular, a variety of data from single-cell sequencing of tumours and normal cells has shown tumours contain only a few copy number states, without a large number of intermediates, suggesting that large-scale copy number changes appear to occur in a punctuated fashion rather than accumulating gradually over time [8]. A limitation of studying this question in bulk sequencing is that detecting subclonal copy number changes with high sensitivity is difficult, particularly in specimens with CIN and suboptimal purity.

### Treatment Resistance

These cases present a unique opportunity to study clonal evolution during therapy. Breast cancer is the prototypical “treatable” solid tumour for which patients receive multiple lines of therapy that usually result in a partial response or stable disease.

There was clear evidence that treatment alters subclonal structure. Disease at autopsy showed subclones that contained mutations conferring chemotherapy resistance. In ER1 one such mutation, TYMS V106M, is at a highly conserved residue in the binding site of thymidylate synthase, adjacent to two mutations known to cause resistance to 5FU in vitro (Fig 2B, yellow branch) [45].

Mutations in the ligand binding domain of *ESR1* have recently been discovered as a common mechanism of resistance to endocrine therapy in metastatic breast cancer [46]. ER2 displayed three different *ESR1* mutations all in different subclones. Furthermore, an *ESR1* S463P mutation occurred in a subclone that already harboured an *ESR1* E380Q, presumably in a different allele. As *ESR1* mutations can be treated with alternative endocrine therapy and represent a tractable form of endocrine resistance, identifying them in patients failing endocrine therapy is of some importance [47]. ER2 had a liver biopsy while alive that did not show any *ESR1* mutations, highlighting the difficulty in managing advanced heterogeneous disease. At the time of the liver biopsy, circulating cell-free DNA in plasma showed detectable low levels of all three *ESR1* mutations (Fig 6F), which increased dramatically by the time of autopsy.

ER3 demonstrated discordant clinical responses on several occasions, with disease in the lung responding while disease in the liver progressed and left and right hilar lymph nodes responding differentially to chemotherapy. Fig 5C shows the divergent subclonal structure of lung, liver, and hilar lymph nodes, although this may be a consequence of heterogeneous treatment responses rather than the cause. Fig 7 also shows that differences in copy number can be discerned between sample pairs in ER3, more so than the other cases, as discussed in the previous section.

**Fig 8:**
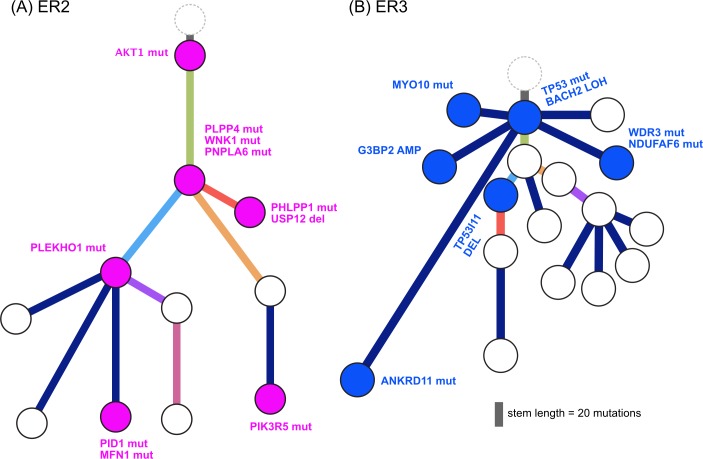
**Evolution of augmented oncogenic signalling in (A) ER2 and (B) ER3.** Subclonal phylogenies with private subclones are displayed with alterations that are expected to modulate oncogenic signalling.

We found additional evidence of this effect when considering the evolutionary trajectory in the context of dominant oncogenic drivers. In ER2 and ER3, there were multiple subclones with alterations in genes that interact with the truncal drivers in each case, *AKT1* and *TP53*, respectively (Fig 8). For ER2, alterations occurred in genes involved in the regulation of phosphatidylinositide phohsphates and PI3K signalling (*PLPP4*, *PNPLA6*, and *PIK3R5*), negative regulators of *AKT1* signalling (*PLEKHO1*, *PHLPP1*, *PID1*, and *USP12*), and downstream effectors of *AKT1* (MFN1). For ER3, alterations occurred in coactivators and facilitators of TP53 activity (*BACH2*, *ANKRD11*, *TP53I11*, *MYO10*, *WDR3*, and *NDUFAF6*), negative regulators of TP53 function (*G3BP2*), and downstream effectors (*CD82*). These findings suggest that resistance to chemotherapy agents may arise from augmentation of existing oncogenic or tumour suppressor signalling pathways rather than direct resistance through altered drug targets or drug metabolism. This could be one explanation why increasing lines of treatment become progressively less effective, despite having non-overlapping mechanisms of action.

### Mutational Signatures Drive Heterogeneity and Treatment Resistance

Due to relatively low numbers of mutations, clear mutational signatures could be established only in ER2 and TN1. The pattern of mutational signatures in these two cases were robust and maintained a stable prevalence even when the starting mutation pool was down sampled to 10% of the total.

TN1 had an unusual composition of mutational signatures (Fig 6A–6C). There was a strong signal from signature 17 in the COSMIC classification [38,39]. This signature has been reported in breast cancer as well as oesophageal, liver, lung, and stomach cancers, melanoma, and B-cell lymphoma [38]. The aetiology is unknown. As signature 17 produces a characteristic T > G transversion that has relatively little overlap with other signatures, the contribution of the signature to the mutations in subclones can be tracked. Mutations arising from this signature were found primarily in metastatic lesions from autopsy, comprising between 9%–45% of the private mutations in four spatially distinct lesions in the lung and liver. The signature was not active in the primary lesions or premortem liver biopsy. These private signature 17 mutations have subclonal fractions in the 0.2–0.6 range and cluster with other mutations to form private subclones. Assuming that it is extremely unlikely a mutational process could cause the same mutation in multiple cells within a single lesion, each private subclone with a relatively high subclonal fraction of the signature 17 mutations must have arisen from a very restricted population of single cells that expanded in each lesion. In addition, as a mutational process per se confers no proliferative advantage, the prevalence of this signature may represent cells that were able to survive a treatment bottleneck, sustaining DNA damage in the process. The only treatment the patient received after the liver biopsy (which did not contain signature 17) was a pan aurora kinase inhibitor. There was no evidence of response to this therapy. It is unclear how an aurora kinase inhibitor could cause this mutational signature. Signatures 24 and 29 also show activity in TN1 samples. Signature 29 has been associated with the mutagenic effect of tobacco chewing on the oral mucosa, and, although distinct from the classic smoking associated signature 4, there is a high degree of overlap. Signature 24 is reported to occur with aflatoxin exposure. The significance of these carcinogen-induced signatures in a triple negative breast cancer is unclear.

ER2 was dominated by signatures 2 and 13 in the COSMIC classification [39], which both relate to the well-known phenomenon of APOBEC deaminase activity (Fig 6D). This signature was present consistently in both early and late clones and allows us to study how APOBEC may contribute to the evolution of breast cancer. Key driver mutations *AKT1* E17K, *SPEN*, and the *ESR1* E380Q mutation are consistent with those induced by APOBEC. However, the *ARID1A* and the *TP53* truncating mutations have a low probability of arising from the APOBEC signature, and the *ESR1* S463P and D538G mutations are not consistent with APOBEC. In this case, therefore, APOBEC-related activity alone was able to furnish most of the putative driver mutations and resulted in an *ESR1* mutation that causes resistance to endocrine therapy but could not explain all of the *ESR1* mutations.

## Discussion

In this study, we analyse four cases chosen to represent difficult clinical scenarios in the contemporary management of advanced breast cancer. We took the approach of extensively sampling metastatic disease at the time of autopsy, performing whole exome sequencing and SNP arrays coupled with high depth validation of discovered mutations. We also analysed samples taken from the primary tumour and, where available, metastatic lesions biopsied whilst patients were still alive. In line with other work utilising the CASCADE program, we found studying advanced disease at the time of death to be highly informative [48]. We found significant heterogeneity present across multiple metastatic sites, and by performing subclonal inference, it was possible to understand the key processes that drive tumour evolution over time. We have shown several novel findings, including how treatment shapes clonal evolution, the importance of mutational processes over a disease course, and augmentation of oncogenic signalling as a mechanism of treatment failure.

Late relapse is a significant problem for ER-positive disease, which continues to show a decline in survival beyond 10 years from diagnosis. In case ER1 with a long clinical latency, we observed that the subclone giving rise to metastatic disease was not detectable at diagnosis with standard sequencing and sampling measures. This has important implications for genomic determinism, or the expectation that genomic assays from a single time point are able to predict clinical outcome and guide therapy. In this case, additional oncogenic drivers from mutations and CNA accumulated before metastatic disease occurred. The long time to recurrence may be because micrometastatic disease remained dormant until acquiring additional drivers, or that the intermediate clone was present early in the natural history but was suppressed by tamoxifen. Although these possibilities cannot be distinguished here, other studies have found evidence that the primary tumour population contains low-frequency subclones that may give rise to metastatic or recurrent disease [49]. Our results are similar to those obtained in another case of recurrent disease after a long latency in lobular breast cancer [1]. Assuming a model in which infrequent subclones may give rise to eventual relapse 5 years or more after initial therapy, prolonging adjuvant endocrine therapy is expected to be beneficial, as has been shown in clinical trials [50,51]. Detecting and eliminating these rare subclones is a difficult proposition, but targeting known clonal driver alterations could be one strategy. These driver alterations could also be used to monitor for disease recurrence via ctDNA.

To our knowledge, ER2 is the first case of de novo metastatic ER-positive/HER2-negative disease to undergo longitudinal sampling of metastases and autopsy along with ctDNA assessments. In contrast to ER1, ER2 showed less divergence between the primary and metastatic lesions. The most likely explanation for this pattern is that metastatic potential was available early in the evolutionary history of this case. Another explanation for this could be reseeding of the primary tumours from a metastatic niche, as has been reported elsewhere [52]. This case did display subclonal patterns consistent with metastatic reseeding between para-aortic nodal sites. That this mechanism exists in breast cancer highlights the difficulties in accurately profiling the cancer genome, because even a lesion once biopsied may significantly change its subclonal structure. Circulating tumour DNA is one method to avoid sampling bias, as was illustrated in this case, in which biopsy of a liver lesion did not reveal any of the three *ESR1* mutations that were detectable in plasma at the time.

In addition to ESR1, all three ER-positive cases showed alterations in *SPEN* after exposure to endocrine therapy. *SPEN* has recently been implicated as a novel tumour suppressor that regulates cell proliferation and inhibits oestrogen receptor downstream signalling, with a role in resistance to tamoxifen [53]. The truncal location of *SPEN* alterations in three different cases suggests this is a bona fide tumour suppressor and mediator of resistance to endocrine therapy.

In ER3, there was the unexpected finding that the earliest subclone was able to metastasise to the ovary. This case is also notable for the widespread copy number derangement that was present in all samples, and, similar to TN1, this may have conferred the metastatic phenotype. ER3 displayed ongoing copy number changes that segregated with subclones in our analysis. This was in contrast to the other ER-positive cases, in which mutational processes dominated. The presence of CIN has been shown in vitro to be associated with drug resistance, and there is some evidence that aneuploidy is an adaptive response in lower eukaryotes [54,55]. There is increasing evidence for punctuated evolution of copy number in cancers [8,56]. This fits well with our data for ER1, ER2, and TN1. The situation for ER3 is less clear cut, as there is ongoing acquisition of copy number changes beyond increases in ploidy. In some cancers, therefore, “active” CIN is likely to be an important mechanism driving evolution, treatment resistance, and heterogeneity. Notably, during treatment with tamoxifen and chemotherapy, ER3 showed discordant responses between lesions in liver and lung. This could be explained by the significant copy number heterogeneity seen in this case, although it is not possible to trace a premortem lesion on imaging to a lesion at autopsy.

Understanding mutational signatures provided valuable insights for ER2 and TN1. In TN1, the late emergence of signature 17 implies rapid rescaling of the subclonal structure of several metastatic lesions. The aetiology of signature 17 is unknown, but the activity of this signature in unrelated subclones at different anatomical sites suggests it can be chemically induced. Furthermore, the presence of apparent carcinogen-induced mutational signatures in TN1 also raises questions about the aetiology of aggressive triple negative breast cancers. In ER2, APOBEC was responsible for many important alterations of functional significance, including the key founding driver mutation. It has been previously noted that APOBEC may give rise to clonal and subclonal driver mutations [57,58], but we demonstrate here that this mutational activity is maintained during treatment and throughout the natural history of the disease. Arresting the activity of APOBEC may be a potential strategy to restrain progression and evolution of APOBEC-enriched cancers.

We identified a potentially novel mechanism of broad treatment resistance to chemotherapy, which arises from augmentation of existing oncogenic signalling. This implies that oncogenic signalling remains essential even in heavily pretreated disease and raises the possibility that combining targeted therapies with chemotherapy may severely restrict the fitness landscape that a tumour can access to achieve treatment resistance. The superior efficacy of such combination therapy is well known, and this approach has been used with great success in HER2-positive breast cancer, for example [59]. Other studies have shown that driver alterations influence response to chemotherapy [7]. Our findings extend these concepts to define augmented oncogenic signalling as a resistance mechanism that is widespread in advanced disease and may be therapeutically tractable.

There are several limitations to this study. WES alone may result in poor resolution to detect subclones, which could underestimate the subclonal diversity present. In contrast to mutations, detection of subclonal copy number events remains difficult, and important subclonal amplifications or deletions may have been missed. Newer technologies, such as single-cell approaches or long read sequencing, will be required to overcome these limitations. In addition, we did not analyse structural variants, the transcriptome, the epigenome, or the proteome, which along with noncoding elements such as long noncoding RNAs or microRNAs could make important contributions to subclonal evolution and phenotypic diversity not captured by WES. Although we have analysed a small number of cases in detail, it is unclear whether our findings are representative of the broader patient population.

In conclusion, we demonstrate the feasibility and value of subclonal inference in understanding the biology and evolutionary history of lethal breast cancer. This approach also provided insight into difficult clinical scenarios in breast cancer. Extension of the whole exome approach to whole genome, transcriptome, and methylome studies, as well as more novel single-cell and long read sequencing technology, is expected to provide further insights, particularly when used in the setting of rapid autopsy studies, which afford the unprecedented ability to sample multiple evolutionary trajectories comprehensively. It is notable that each case studied was unique in the processes that ultimately resulted in death. It is unclear if patterns will be found that can generalize across patient subgroups: for this, we will need large cohorts for which we can track genomic evolution from diagnosis to death. To that end, our prospective rapid autopsy program, which continues to accrue in breast cancer and other cancer types [9], as well as other international efforts will be essential to help us understand how cancer disseminates and ultimately becomes resistant to treatment [60].

## Supporting Information

S1 Fig Immunohistochemistry for the protein product of the *CDH4* gene, which contains a truncal mutation in TN1. Both primary tumour and liver metastases show evidence of expression.
(TIF)

S1 Table Description of samples. (XLSX)

S2 Table Quality control metrics for sequencing. (XLSX)

S3 Table Validation rates. (XLSX)

S1 Text STROBE Checklist. (DOC)

## Abbreviations

ABC: avidin-biotin-complex
CASCADE: prospective community-based rapid autopsy program
CNA: copy number alterations
CIN: chromosomal instability
ctDNA: circulating DNA
ER: oestrogen receptor
FFPE: formalin-fixed, paraffin-embedded
HER2: human epidermal growth factor receptor 2
kConFab: Kathleen Cuningham Foundation Consortium for Research into Familial Breast Cancer
LOH: loss of heterozygosity
SNP: single nucleotide polymorphism
SNV: single nucleotide variant
WES: whole exome sequencing
