# Correction: The Subclonal Architecture of Metastatic Breast Cancer: Results from a Prospective Community-Based Rapid Autopsy Program "CASCADE"

**DOI:** 10.1371/journal.pmed.1002302

**Published:** 2017-04-21

**Authors:** Peter Savas, Zhi Ling Teo, Christophe Lefevre, Christoffer Flensburg, Franco Caramia, Kathryn Alsop, Mariam Mansour, Prudence A. Francis, Heather A. Thorne, Maria Joao Silva, Nnennaya Kanu, Michelle Dietzen, Andrew Rowan, Maik Kschischo, Stephen Fox, David D. Bowtell, Sarah-Jane Dawson, Terence P. Speed, Charles Swanton, Sherene Loi

There is an error in reference 35. The correct reference is: Niknafs N, Beleva-Guthrie V, Naiman DQ, Karchin R. SubClonal Hierarchy Inference from Somatic Mutations: Automatic Reconstruction of Cancer Evolutionary Trees from Multi-region Next Generation Sequencing. PLoS Comput Biol. 2015;11(10): e1004416. doi:10.1371/journal.pcbi.1004416. PMID: 26436540.
