# Supplementary figures and images for: The Subclonal Architecture of Metastatic Breast Cancer: Results from a Prospective Community-Based Rapid Autopsy Program “CASCADE”
Source: PLoS Med. 2016 Dec 27;13(12):e1002204. doi: 10.1371/journal.pmed.1002204 (PMC5189956; doi:10.1371/journal.pmed.1002204)

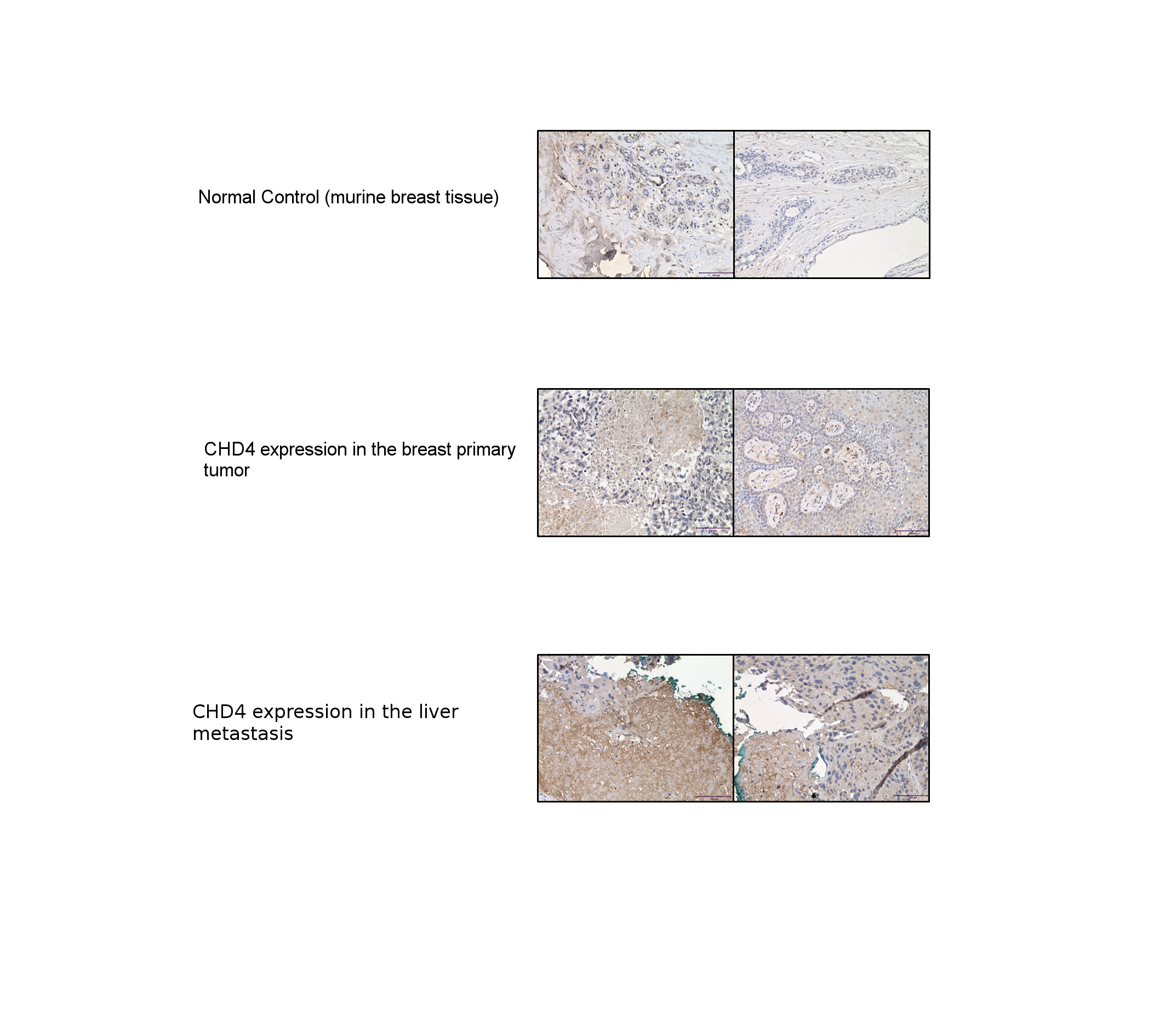

Supplement: S1 Fig — Both primary tumour and liver metastases show evidence of expression. (TIF) [file pmed.1002204.s001.tif]
