# Supplementary material for: The Subclonal Architecture of Metastatic Breast Cancer: Results from a Prospective Community-Based Rapid Autopsy Program “CASCADE”
Source: PLoS Med. 2016 Dec 27;13(12):e1002204. doi: 10.1371/journal.pmed.1002204 (PMC5189956; doi:10.1371/journal.pmed.1002204)
Supplement: S1 Text — (DOC) [file pmed.1002204.s005.doc]

STROBE Statement—checklist of items that should be included in reports of observational studies

|  | | Item No | Recommendation |
| --- | --- | --- | --- |
| **Title and abstract** | | 1 | *(*a) Indicate the study’s design with a commonly used term in the title or the abstract  **Response:** The title indicates this was a prospective study. |
|  |  |  | (*b*) Provide in the abstract an informative and balanced summary of what was done and what was found  **Respone:** The salient points of the methods and analysis and the key findings are presented, along with an indication of the limitations. |
| Introduction | | | |
| Background/rationale | | 2 | Explain the scientific background and rationale for the investigation being reported  **Response:** The context and prior work are summarised in the introduction. |
| Objectives | | 3 | State specific objectives, including any prespecified hypotheses  **Response:** Our objectives are described in the final paragraph of the introduction. |
| Methods | | | |
| Study design | | 4 | Present key elements of study design early in the paper  **Response:** The key analytical methods and recruitment processes are in the abstract, introduction and early in the methods. |
| Setting | | 5 | Describe the setting, locations, and relevant dates, including periods of recruitment, exposure, follow-up, and data collection  **Response:** This is discussed in the methods section. |
| Participants | | 6 | (*a*) *Cohort study*—Give the eligibility criteria, and the sources and methods of selection of participants. Describe methods of follow-up  *Case-control study*—Give the eligibility criteria, and the sources and methods of case ascertainment and control selection. Give the rationale for the choice of cases and controls  *Cross-sectional study*—Give the eligibility criteria, and the sources and methods of selection of participants |
|  |  |  | (*b*) *Cohort study*—For matched studies, give matching criteria and number of exposed and unexposed  *Case-control study*—For matched studies, give matching criteria and the number of controls per case  **Response:** This being an autopsy study, these study designs do not apply. The eligibility criteria for recruitment are explained. |
| Variables | | 7 | Clearly define all outcomes, exposures, predictors, potential confounders, and effect modifiers. Give diagnostic criteria, if applicable  **Response:** A wider variety of genomic variables were considered. Outcomes were qualitative details regarding the patient history, and are discussed as relevant. |
| Data sources/ measurement | | 8* | For each variable of interest, give sources of data and details of methods of assessment (measurement). Describe comparability of assessment methods if there is more than one group  **Response:** This is evident in the methods and results. |
| Bias | | 9 | Describe any efforts to address potential sources of bias  **Response:** The potential for bias is discussed in the limitations section. Several techniques to avoid bias are mentioned in the methods section, as they relate to the relevant analysis method. |
| Study size | | 10 | Explain how the study size was arrived at  **Response:** As this was an exploratory study, power calculations are not possible. |
| Quantitative variables | | 11 | Explain how quantitative variables were handled in the analyses. If applicable, describe which groupings were chosen and why  **Response:** Standard methods for analysing genomic data are described. |
| Statistical methods | | 12 | (*a*) Describe all statistical methods, including those used to control for confounding |
|  |  |  | (*b*) Describe any methods used to examine subgroups and interactions |
|  |  |  | (*c*) Explain how missing data were addressed |
|  |  |  | (*d*) *Cohort study*—If applicable, explain how loss to follow-up was addressed  *Case-control study*—If applicable, explain how matching of cases and controls was addressed  *Cross-sectional study*—If applicable, describe analytical methods taking account of sampling strategy |
|  |  |  | (*e*) Describe any sensitivity analyses  **Response:** This is addressed in the methods section as necessary. Hypothesis based statistical tests are not relevant to this study. |
| Results | | | |
| Participants | 13* | (a) Report numbers of individuals at each stage of study—eg numbers potentially eligible, examined for eligibility, confirmed eligible, included in the study, completing follow-up, and analysed | |
|  |  | (b) Give reasons for non-participation at each stage | |
|  |  | (c) Consider use of a flow diagram  **Response:** the number of samples, the most relevant metric here, is described and tabulated (supplementary information). | |
| Descriptive data | 14* | (a) Give characteristics of study participants (eg demographic, clinical, social) and information on exposures and potential confounders | |
|  |  | (b) Indicate number of participants with missing data for each variable of interest | |
|  |  | (c) *Cohort study*—Summarise follow-up time (eg, average and total amount)  **Response:** The cases histories are described. | |
| Outcome data | 15* | *Cohort study*—Report numbers of outcome events or summary measures over time | |
|  |  | *Case-control study—*Report numbers in each exposure category, or summary measures of exposure | |
|  |  | *Cross-sectional study—*Report numbers of outcome events or summary measures  **Response:** This is not relevant to our study. | |
| Main results | 16 | (*a*) Give unadjusted estimates and, if applicable, confounder-adjusted estimates and their precision (eg, 95% confidence interval). Make clear which confounders were adjusted for and why they were included | |
|  |  | (*b*) Report category boundaries when continuous variables were categorized | |
|  |  | (*c*) If relevant, consider translating estimates of relative risk into absolute risk for a meaningful time period  **Response:** Not relevant | |
| Other analyses | 17 | Report other analyses done—eg analyses of subgroups and interactions, and sensitivity analyses  **Response:** The analyses are described in detail in the Methods, and also diagrammed in figure 1. | |
| Discussion | | | |
| Key results | 18 | Summarise key results with reference to study objectives  **Response:** This is in the discussion section. | |
| Limitations | 19 | Discuss limitations of the study, taking into account sources of potential bias or imprecision. Discuss both direction and magnitude of any potential bias  **Response:** The limitations are described. | |
| Interpretation | 20 | Give a cautious overall interpretation of results considering objectives, limitations, multiplicity of analyses, results from similar studies, and other relevant evidence  **Response:** A balanced interpretation with attention to the necessary caveats is provided. | |
| Generalisability | 21 | Discuss the generalisability (external validity) of the study results  **Response:** This is discussed. | |
| Other information | | | |
| Funding | 22 | Give the source of funding and the role of the funders for the present study and, if applicable, for the original study on which the present article is based  **Response:** This is provided. | |

*Give information separately for cases and controls in case-control studies and, if applicable, for exposed and unexposed groups in cohort and cross-sectional studies.

**Note:** An Explanation and Elaboration article discusses each checklist item and gives methodological background and published examples of transparent reporting. The STROBE checklist is best used in conjunction with this article (freely available on the Web sites of PLoS Medicine at http://www.plosmedicine.org/, Annals of Internal Medicine at http://www.annals.org/, and Epidemiology at http://www.epidem.com/). Information on the STROBE Initiative is available at www.strobe-statement.org.
